# Vascular Endothelial Growth Factor Variants (936C/T, 634C/G, 2578A/C) and Their Genotype–Haplotype Association with Recurrent Implantation Failure in Infertile Women: A Single-Center Analytical Study

**DOI:** 10.3390/diagnostics15070868

**Published:** 2025-03-28

**Authors:** Lucia Maria Procopciuc, Mihaela Iancu, Gabriela Valentina Caracostea, Iulian Goidescu, Adelina Staicu, Roxana Liana Lucaciu, Adriana Corina Hangan, Sidonia Gog Bogdan, Mihai Surcel

**Affiliations:** 1 Department of Medical Biochemistry, “Iuliu-Hațieganu” University of Medicine and Pharmacy, 400349 Cluj-Napoca, Romania; lprocopciuc@umfcluj.ro; 2 Medical Informatics and Biostatistics, Department 1, Faculty of Nursing and Health Sciences, “Iuliu-Hațieganu” University of Medicine and Pharmacy, 400349 Cluj-Napoca, Romania; 3 Medicover Hospital, 407062 Suceagu, Romania; caracostea1@yahoo.com; 4 Department of Obstetrics and Gynecology, “Iuliu-Hațieganu” University of Medicine and Pharmacy, 400015 Cluj-Napoca, Romania; iuliangoidescu@gmail.com (I.G.); adelina.staicu@umfcluj.ro (A.S.); mihai_surcel@yahoo.com (M.S.); 5 Department of Pharmaceutical Biochemistry and Clinical Laboratory, Faculty of Pharmacy, “Iuliu-Hațieganu” University of Medicine and Pharmacy, 400012 Cluj-Napoca, Romania; liana.lucaciu@umfcluj.ro; 6 Department of Inorganic Chemistry, Faculty of Pharmacy, “Iuliu-Hațieganu” University of Medicine and Pharmacy, 400012 Cluj-Napoca, Romania; adriana.hangan@umfcluj.ro; 7 Department of Sugery and ATI, Faculty of Veterinary Medicine, University of Agricultural Sciences and Veterinary Medicine, 400372 Cluj-Napoca, Romania; sidonia.bogdan@usamvcluj.ro

**Keywords:** recurrent implantation failure, VEGF-*936C/T*, VEFG-*634C/G*, VEGF-*2578C/A*, haplotype analysis

## Abstract

**Background:** Vascular Endothelial Growth Factor (VEGF) is a key regulator in angiogenesis and contributes to a successful implantation. The current study has the following objective: to perform genotyping and haplotyping analysis to confirm whether the VEGF-*936C/T*, VEGF-*634C/G*, and VEGF-*2578C/A* gene polymorphisms are associated with the susceptibility for recurrent implantation failure (RIF) in Romanian females at reproductive age. **Materials and Methods:** In total, 41 infertile women experiencing recurrent implantation failure and 44 women with minor infertility were genotyped for VEGF polymorphisms using PCR-RFLP analysis. **Results:** The VEGF-*936C/T* polymorphism in the dominant model, (C/T+T/T), represents an increased risk factor for recurrent implantation failure, the odds being 2.70 (95% CI: [1.04, 7.00]). Also, VEGF-*2578C/A* gene polymorphism represents the risk factor of RIF under the codominant (adjusted-OR = 5.28, 95% CI: [1.42, 19.65]) and recessive models (adjusted-OR = 5.15, 95% CI: [1.55, 17.09]). Patients carrying the VEGF-*T936* allele or VEGF-*C2578* allele had 2.25-fold and 2.36-fold increased odds of implantation failure (95% CI: [1.05, 4.81], *p* = 0.034) and 95% CI: [1.27, 4.39], *p* = 0.006), respectively. The results of the haplotype-based regression analysis reveal that patient carriers of the VEGF-*936/-634/-2578 T-C-A* haplotype had 12.39 increased odds of RIF. Also, carriers of the VEGF-*936/-2578 T-A* haplotype had 9.56-fold (*p* = 0.0113) increased odds of RIF after adjusting for age. **Conclusions:** We found a significant association between VEGF-*936C/T* and VEGF-*2578C/A* polymorphisms and the odds of RIF in this cohort of Romanian infertile women. Haplotype analysis suggested the role of VEGF-*936/-634/-2578 T-C-A* and VEGF-*936/-2578 T-A* haplotypes as a risk factors for RIF.

## 1. Introduction

Recurrent implantation failure (RIF) poses a complex and formidable challenge in the realm of assisted reproduction. While its prevalence remains a topic of debate, the majority of authors agree on an approximate rate of 10% (Ma et al., 2023) [1]. Despite extensive clinical interventions and substantial financial investment, the decreased pregnancy rates associated with RIF evoke feelings of anxiety and despair among couples undergoing this procedure, while also inducing frustration among healthcare providers. The European Society of Human Reproduction and Embryology’s preimplantation genetic diagnosis consortium has defined this condition as the inability to achieve a clinical pregnancy after more than three transfers of high-quality embryos or ten embryos in multiple transfer cycles (Cimadomo et al., 2023) [2]. The primary explanation for this situation lies in the intricate nature of recurrent implantation failure, which encompasses a multitude of etiologies. When contrasted with typical IVF patients, the endometrium stands out as a notably significant factor, in addition to potential paternal or oocyte quality concerns (Cimadomo et al., 2021) [3]. Endometrium regeneration is governed by a myriad of molecular and cellular interactions, regulated by endocrine factors, locally produced steroids, immune factors, angiogenetic factors, and factors associated with uterine microbiota (Critchley et al., 2020) [4].

There are many risk factors of RIF, such as maternal age, body mass index (BMI), tobacco, alcohol intake, immunological factors, and abnormal angiogenesis (Bashiri et al., 2018; Guo et al., 2021) [5,6]. The quality of embryos used for in vitro fertilization (IVF) is influenced by maternal age. The frequency of aneuploidy increases as the maternal age increase. An increased maternal age influences a decrease in pregnancy rates (Zeadna et al., 2015) [7]. Higher rates of implantation were observed in patients younger than 35 years than in patients older than 44 years [8].

Normal weight is a favorable factor for increasing the chance of IVF. Moragianni et al. (2012) found that the risk of implantation failure is higher in obese patients (BMI > 30 kg/m^2^). Women with BMI higher than 40 kg/m^2^ had higher rates of miscarriage [9]. Obesity could also affect oocyte quality. Orvieto et al. (2009) compared the risk of RIF in obese women and women of normal weight and found an risk increased in obese women, even though both categories had the same number of collected oocytes [10]. Smoking influences the successful of implantation of the embryo and also the estradiol levels during ovarian stimulation (Cnattingius et al., 2004) [11]. Waylen et al. (2009) suggested that smoking influences pregnancy outcome, with smoking women having decreased life birth rate [12]. Moreover, the toxic effect of nicotine and carbon monoxide is related to a depletion in oxygen to the fetus, and also the vasoconstriction determined by nicotine influences the nutrient intake to the fetus. Smoking has an effect not just on female, but also on male fertility, influencing decreased sperm count and motility (Kunzle et al., 2003) [13]. Generally, alcohol intake negatively influences pregnancy outcome. It affects neurocognitive function, so it is recommended to be avoided during pregnancy. For the successful implantation, trophoblast invasion activates the maternal immune response to fetal antigens, local immune cells promoting placental development. In this stage, there is an activation of immune cells such as innate lymphocytes, T cells, decidual dendritic cells, and macrophages, processes associated with RIF (Li et al., 2021) [14].

There could also be other risk factors for RIF, such as embryo factors caused by genetic abnormalities and uterine factors caused by anatomical abnormalities (Mrozikiewicz et al., 2021; Busnelli et al., 2021) [15,16]. Such factors include the low quality of gametes in older women, thrombophilias (i.e., factor V Leiden), uterine factors (i.e., congenital uterine anomalies, endometrial polyps, submucosal fibroids, intrauterine adhesions), and adnexal pathologies (i.e., hydrosalpinx) (Cakiroglu et al., 2020) (Sheikhansari et al., 2020) [17,18].

Recent studies on patients with recurrent implantation failure (RIF) have unveiled evidence of molecular disruptions occurring at the endometrial level associated with vasoactive factors (Cimadomo et al., 2021) [3].

Endometrial angiogenesis is regulated by a complex network of signaling molecules and receptors, one of the most important factors which promote vasculogenesis and angiogenesis in the placenta, predominantly belonging to the Vascular Endothelial Growth Factor (VEGF) family, also named the vasculotropine or vascular permeability factor, and its splicing variants (Ghalehbandi et al., 2023) [19]. Sugino et al. (2002) found that endometrial VEGF increases in the luteal phase. In the meantime, sflT1 decreases [20].

For a successful embryo implantation, increased vascular permeability and endothelial cell proliferation within the endometrium has been shown to be necessary (Rowe et al., 2003) [10,21]. The study performed by Kapiteijn et al. (2006) [22] in humans showed that implantation failure is associated with inadequate angiogenesis. Successful pregnancy requires the angiogenesis of the endometrium, and VEGF represents one of the key regulator of this process.

In humans, the VEGF family comprises four isoforms: VEGFA, VEGFB, VEGFC, and VEGFD. Moreover, blastocysts express VEGF, thus implanting embryo promotes angiogenesis by binding the VEGF to its endometrial receptors, VEGFR1 (FLT1 gene), VEGFR2 (KDR gene), and VEGFR3 (FLT4 gene). sVEGFR-1 (sFlt-1) represents a soluble form of VEGFR-1 and can bind VEGF, VEGF-B, and PlGF, preventing the binding of these molecules to their receptors [23]. So, sFlt1 has an antiangiogenic effect. Edgel et al. (2018) suggested that sflT1 activity correlates with unexplained infertility [24]. PlGF, with lower affinity for VEGFR-1 than VEGF, could potentiate the angiogenic effect of VEGF [25].

Also, these receptors have a role in vascular permeability and lymphangiogenesis, all of these processes have a role in maintaining successful pregnancy. There are increased intrauterine VEGF concentration in women with infertility during the late secretory and premenstrual phases (Alitalo et al., 2002; Guo et al., 2021l; Kruessel et al., 2001; Goodman et al., 2008) [6,26,27,28].

Bansal et al. (2017) shows that VEGF levels are increased in women with RIF, as compared to fertile women [29]. The study performed by Chen et al. (2017) suggested increased VEGF expression in the endometrium during embryo implantation [30]. Gao et al. (2015) [31] suggested increased activated NK cells in women with RIF. This is probably an explanation for increased VEGF levels, because NK cells express and release VEGF. In the same study, he compared women with RIF and fertile women and found that VEGF-A, VEGF-C, and PLGF decreased in the glandular epithelium, luminal epithelium, and stroma in women with RIF than in fertile women [31].

Moreover, PIGF levels are higher in the endometrium of women with successful embryo implantation compared to those with RIF (Santi et al., 2011) [32]. The upregulation of VEGF and downregulation of sFlt1 in the lutheal phase increase endometrial angiogenesis, suggesting the role of angiogenesis in this process, and prepare the endometrium for embryo implantation (Conttrell et al., 2017) [33].

When considering the impact of specific Vascular Endothelial Growth Factor (VEGF) polymorphisms on pathology, a wide array of conditions have been documented. Viewed through the lens of gynecological practice, significant concerns arise regarding reproductive challenges, such as recurrent implantation failure (RIF) or recurrent miscarriage (RM), as well as conditions like Polycystic Ovary Syndrome (PCOS) or endometriosis (Zeng et al., 2021; Jung et al.,2018; Boudjenah et al., 2012; Gupta et al., 2019; Zhao et al., 2020; Liu et al., 2016) [34,35,36,37,38,39].

The VEGF gene is located on chromosome 6p21.3 and has 8 exons and 7 introns. Different polymorphisms located in the VEGF gene, such as -*2578C/A* (rs 699947), -*634C/G* (rs 2010963), and +*936C/T* (rs 3025039), were associated with protein expression, VEGF levels, and also VEGF activity (Turienzo et al., 2020) [40]. Kim et al. (2011) showed that the VEGF-*936C/T* polymorphism (located in the 3′ end of the promoter region of the gene-UTR) is associated with RNA splicing decreasing the VEGF levels [41]. The VEGF-*2578C>A* variant is located in the promoter region of the VEGF gene, the *CC2578* genotype being associated with increased VEGF secretion. The VEGF-*634C>G* variant is located in the 5′UTR of the VEGF gene, associated with increased VEGF expression. The association of this variant with VEGF levels is controversial; some authors say that the *C634* allele is associated with decreased VEGF levels, whereas others say that the *G634* allele has this role (Watson et al., 2000; Wongpiyabovorn et al., 2011; Hansen et al., 2010; Awata et al., 2002) [42,43,44,45].

There are studies, such as the one performed by Li et al. (2013), which investigated these polymorphisms in different obstetrical and gynecological diseases [46].

Numerous studies have investigated the association between recurrent implantation failure (RIF) and Vascular Endothelial Growth Factor (VEGF). In a recent meta-analysis, Zeng et al. (2021) identified that the VEGF-*2578AA* genotype, -634G allele, and VEGF-*2578/-1154/-634/-936 A-A-G-C* haplotype may serve as genetic markers associated with [34].

Accumulating evidence suggests that eutopic endometrium in women with endometriosis or PCOS exhibits elevated Vascular Endothelial Growth Factor (VEGF) expression (Zhao et al., 2020; Liu et al., 2016) [38,39].

Objectives: (i) To compare the allelic and genotypic distributions of VEGF-*936C/T*, VEGF-*634C/G*, and VEGF-*2578C/A* gene polymorphisms between infertile women with recurrent implantation failure (RIF) and women with minor infertility as a control group; (ii) to analyze haplotype frequencies between infertile women with RIF and the control group; and (iii) to explore the differences in hormone levels in patients carrying variant genotypes of VEGF polymorphisms within each of the studied groups.

## 2. Materials and Methods

### 2.1. Study Design and Participant Recruitment

We conducted a case–control study within the Assisted Reproduction Department of the 1st Obstetrics and Gynecology Clinic (Cluj-Napoca, Romania) between May 2023 and January 2024. The study group consisted of 41 infertile women experiencing recurrent implantation failure, defined as individuals who had undergone a minimum of three fresh or frozen cycles and had received at least four high-quality blastocysts, yet failed to achieve pregnancy, as evidenced by human chorionic gonadotropin (hCG) levels below 5 U/mL. Women were excluded from the study if they met any of the following criteria: age exceeding 40 years, presence of untreated hydrosalpinx confirmed by imaging, moderate or severe endometriosis, diagnosed inherited thrombophilia or antiphospholipid syndrome, abnormal chromosome(s) identified in either or both partners, a body mass index > 35 kg/m^2^, or congenital uterine anomalies.

According to our study design, the control group comprised 44 infertile women referred to our center, or who self-referred, with primary infertility causes unlikely to be related to endometrial issues. They are women with minor infertility, ensuring that any major endometrial or follicular pathologies were excluded. To ensure this, we conducted thorough clinical examinations, which included detailed medical history assessments, hysterosalpingography, sperm analysis, and hormonal assessments. Exclusion criteria for the control group were as follows: individuals over the age of 40, presence of severe hydrosalpinx confirmed by imaging, moderate or severe endometriosis, diagnosed inherited thrombophilia or antiphospholipid syndrome, a body mass index exceeding 35 kg/m^2^, congenital uterine anomalies, or severe oligospermia.

Subsequently, these individuals were offered a range of interventions, including surgical procedures like neo-salpingostomy or tubal reversion, as well as ovulation induction, timed intercourse, or intrauterine insemination. Inclusion in the control group was contingent upon successful pregnancy achievement, with a minimum gestational age of 12 weeks serving as the criterion. Patients who did not obtain and maintain the pregnancy were excluded from this study.

All patients participating in this study were provided with detailed information regarding the study’s objectives and methodology. Prior to their involvement, informed consent was obtained from each participant. Additionally, medical history data were meticulously collected from all enrolled participants at the time of study enrollment.

Dysmenorrhea was evaluated using a visual analog scale (VAS) where patients indicated their worst pain level by making a line on a 10 cm scale. In this study, dysmenorrhea was classified as mild to moderate if the VAS was less than 6, and severe if the VAS was 7 or higher. Menstrual blood loss was assessed using a pictorial blood assessment chart (PBAC). It is a self-administered chart that comprises a visual scoring system that depicts a graded series of soiled tampons and pads. In this study, a PBAC score of 100 or higher was classified as severe menorrhagia.

Each participant underwent a transvaginal ultrasound examination to assess the uterus and adnexa, utilizing a 5–7.5 MHz Medison Accuvix A30 Ultrasound System.

Any focal lesions including polyps, fibroids, intrauterine adhesions (IUAs), and retained products of conception, among others, were meticulously documented. Endometrial thickness (ET) was measured in the mid-sagittal plane and at the point of maximum thickness of the endometrial stripe.

This research was carried out in compliance with the principles outlined in the Declaration of Helsinki and was granted approval by the Ethics Committee of “Iuliu Hatieganu” University of Medicine and Pharmacy (protocol no. 222/10 May 2023).

#### 2.1.1. Hormone Level Determination

In order to determine hormone levels, 2 mL of blood sample were drawn in day 2–3 of the menstrual cycle. Estradiol (E2), follicle-stimulating hormone (FSH), luteinizing hormone (LH), progesterone, and testosterone levels were quantified utilizing a chemiluminescence immunoassay conducted using the Architect analyzer manufactured by Abbott (Abbott Park, IL, USA). The precision of the assay for estradiol was evaluated, revealing the following: an intra-assay coefficient of variation (CV) of 7.4% and an inter-assay CV of 4.5%; for FSH, an intra-assay coefficient of variation of 5.9% and an inter-assay coefficient of variation of 7.9%; for LH, an intra-assay coefficient of variation of 4.1% and an inter-assay coefficient of variation of 4.3%; for progesterone, an intra-assay coefficient of variation of 4.2% and an inter-assay coefficient of variation of 7.1%; for LH, an intra-assay coefficient of variation of 4.1%; and for testosterone, an intra-assay variation of 3.4% and an inter-assay variation of 5.1%. Serum AMH determinations were performed with the electrochemiluminescence immunoassay Vidas. The kits were provided by bioMérieux SA, Marcy-l’Étoile, France, with an intra-assay coefficient of variation of 6.6% and an inter-assay coefficient of variation of 8.3%.

#### 2.1.2. Genetic Methods

VEGF-*936C/T*, VEGF-*634C/G*, and VEGF-*2578C/A* genetic variants were determined using the PCR-RFLP methods described by Papazoglou et al. (2004) and Liu et al. (2009), and they were optimized in our laboratory in the Medical Biochemistry Department of the University of Medicine and Pharmacy, Cluj-Napoca [47,48].

### 2.2. DNA Isolation

DNA was isolated from 5 mL whole blood samples collected into EDTA tubes taken from infertile women with RIF and control women. The DNA extraction was performed using a Zymo research kit (Quick-DNAMiniprep, Kit-Zymo Research Corporation, Freiburg, Germany) according to the manufacturer’s protocol. The samples were stored at −20 °C until genotyping.

### 2.3. PCR Amplification

In order to amplify the interest fragments of 208 bp (VEGF-*936C/T*), 304 bp (VEGF-*634C./G*), and 324 bp (VEGF-*2578C/A*), genotyping was performed using a polymerase chain reaction (PCR). The reactions were carried out using 20 ng DNA template, 200 μM deoxynucleotide triphosphates (dNTP-dATP, dGTP, dCTP, dTTP), a pair of specific primers, different concentrations for each genetic variation, a *Taq polymerase* buffer containing 20 mM Tris-HCl (pH 8.0), 1 mM DTT, 0.1 mM EDTA, 100 mM KCl, 0.5% (*v*/*v*) Nonidet P40, 0.5% (*v*/*v*) Tween20, and 50% (*v*/*v*) glycerol, 2.0 mM MgCl_2_, and 0.625 U *Taq polymerase*. The specific primers provided by the manufacturer—Eurogentec (Kaneka Eurogentec S.A. Biologics Division, Liege, Belgium)—were lyophilized and reconstituted in sterile DDW (100 µM) and stored at −20 °C.

The amplification occurred in an iCycler C1000 BioRad (Bio-Rad Life Science, Hercules, CA, USA), and the PCR amplification protocols were as follows: initial denaturation at 95 °C for 60 s, 34 cycles of denaturation at 95 °C for 10 s, primer annealing for 30 s, primer extension at 72 °C for 20 s, and a final extension for 30 s at 72 °C. The sequence of the specific primers, their concentrations, and also the PCR conditions (primer annealing temperature) are showed in Table 1.

The amplification was checked using electrophoresis of 10 µL amplified PCR product on 2% agarose gel containing 10 mg/mL ethidium bromide. Gel was visualized on a UV transilluminator and photographed.

### 2.4. Restriction Fragment Length Polymorphism (RFLP)

In total, 5 μL of PCRs (208 bp—VEGF *936C/T*, 308 bp—VEGF *634C/G*, 324 bp—VEGF *2578C/A*) were digested with the 2U-specific restriction enzyme for each polymorphism in 10 μL mixture.

The restriction enzymes were obtained from New England Biolabs (New England Biolabs UK, Ltd., Hitchin, UK). The specificity of enzymatic digestion was checked by migration of the samples in 3% agarose gel stained with 10 mg/mL ethidium bromide solution and visualized in UV light.

**Figure 1:**
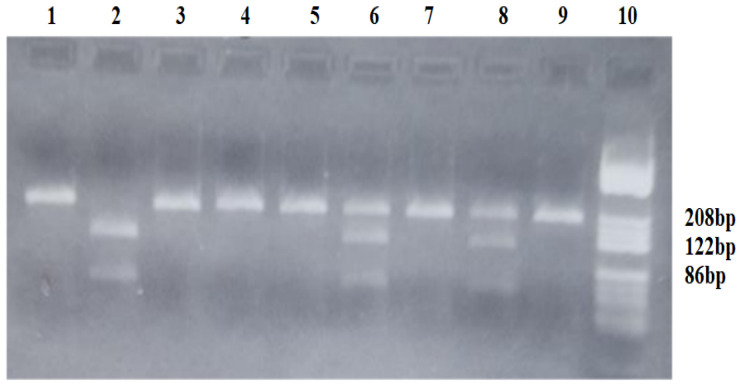
Agarose gel electrophoresis for identification of the VEGF-*936C/T* polymorphism. Lane 10—pBRHaeIII Digest DNA molecular marker V; Lanes 1,3,4,5,7,9—homozygous *CC936* genotype: fragment of 208 bp; Lanes 6,8—heterozygous *CT936* genotype: fragments of 208, 122 and 86 bp; Lane 2—homozygous *TT936* genotype: fragments of 122 and 86 bp.

The VEGF-*936C/T* polymorphism creates a new restriction site for the *NlaIII* enzyme. The *C936* allele was characterized by the presence of an undigested fragment of 208 bp, while the *T936* allele was characterized by two fragments of 122 and 86 bp. Patient carriers of the *CC936* genotype presented one fragment of 208 bp, while those carrying the *CT936* heterozygous genotype presented three fragments of 208, 122, and 86 bp. Carriers of the *TT936* homozygous genotype presented two fragments of 122 and 86 bp. Figure 1 represents the gel picture showing the three genotypes for the VEGF-*936C/T* polymorphism.

**Figure 2:**
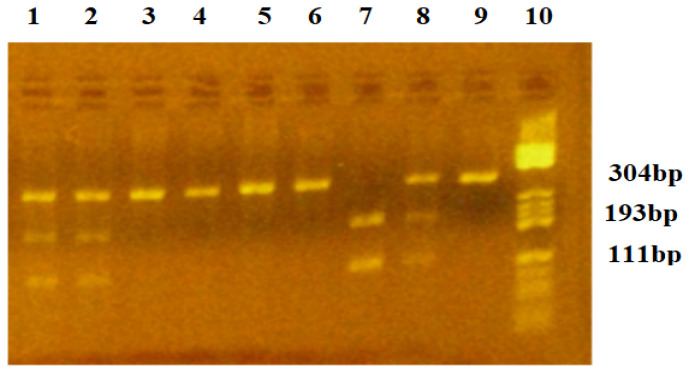
Agarose gel electrophoresis for identification of the VEGF-*634C/G* polymorphism. Lane 10—pBRHaeIII Digest DNA molecular marker V; Lanes 3,4,5,6,9—homozygous *CC634* genotype: fragment of 304 bp; Lane 7—homozygous *GG634* genotype: fragments of 193 and 111 bp; Lanes 1,2,8—heterozygous *CG634* genotype: fragments of 304, 193, 111 bp.

The VEGF-*634C/G* polymorphism abolishes an old site for the *BsmFI* restriction enzyme. The *C634* allele was characterized by an undigested fragment of 304 bp, while the *G634* allele was characterized by two fragments of 193 and 111 bp. Carriers of the *CC634* homozygous genotype presented one fragment of 304 bp. Carriers of the *GG634* genotype presented two fragments of 193 and 111 bp. The *GC634* heterozygous carriers presented three fragments of 304, 193, and 111 bp. Figure 2 represents the gel picture showing the three genotypes for the VEGF-*634G/C* polymorphism.

**Figure 3:**
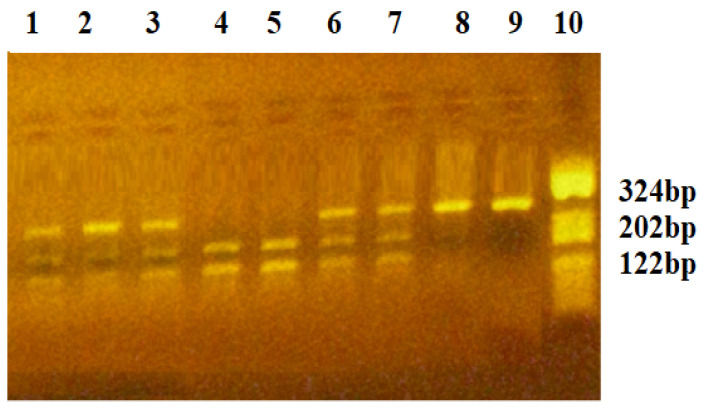
Agarose gel electrophoresis for identification of the VEGF-*2578C/A* polymorphism. Lane 10—pBRHaeIII Digest DNA molecular marker V; Lanes 8,9—homozygous CC2578 genotype: fragment of 324 bp; Lanes 1,2,3,6,7—heterozygous CT2578 genotype: fragments of 324, 202, 122 bp; Lanes 4,5—homozygous AA2578 genotype: fragments of 202, 122 bp.

The VEGF-*2578C/A* polymorphism creates a new restriction site for *BglII* enzyme. The *C2578* allele was characterized using the presence of an undigested 324 bp fragment, while the *A2578* allele was characterized using two fragments of 202 and 122 bp. Patient carriers of the *CC2578* genotype presented one fragment of 324 bp, while those carrying the *CA2578* heterozygous genotype presented three fragments of 324, 202, and 122 bp. Carriers of the *AA2578* homozygous genotype presented two fragments of 202 and 122 bp. Figure 3 presents the gel picture, showing the genotypes of the VEGF-*2578C/A* polymorphism.

Details of PCR-RFLP reagents and conditions for genotyping and enzymatic digestion methods have been given in Table 1.

#### Statistical Analysis

Quantitative continuous characteristics were described using the centrality (dispersion) measures as arithmetic mean (standard deviation) for data with Gaussian distribution or median (interquartile range, IQR = [Q1, Q3], Q1: first quartile; Q3: third quartile). An evaluation of univariate gaussian distribution was conducted using the Shapiro–Wilk test, visual inspection of quantile-quantile plots (Q-Q plots), and descriptive measures of the shape of empirical distribution.

Differences in allele and genotype frequencies of VEGF gene polymorphisms between infertile women with RIF and the non-RIF group were tested using the Chi-square or exact Fisher’s tests. The departure from the Hardy–Weinberg Equilibrium (HWE) for studied SNPs was tested using the exact Chi-square test from “SNPassoc” R package Version 2.1-0 [49].

The association between studied VEGF gene polymorphisms and the odds of RIF was evaluated using unconditional binomial logistic regression under three inheritance genetic models (the codominant, dominant, and recessive models). To control the family-wise error rate, we applied the false discovery rate (FDR)-corrected-*p*-values. The effect size of association was described using the unadjusted odds ratio (OR) with a 95% confidence interval (95% CI) and adjusted odds ratio (OR), with adjustments for the ages of patients.

We also tested the non-random associations of the alleles of VEGF gene polymorphisms at different loci by measures of linkage disequilibrium (LD) as D’ and r^2^ statistics. The haplotype analysis was performed in order to estimate haplotype frequencies using the expectation-maximization algorithm of R-project package “haplo.stats” [50]. The differences in haplotype frequencies between infertile women with RIF and the non-RIF group were tested using an additive generalized linear model.

A comparison of distributions of hormone levels between infertile women with RIF and non-RIF group was performed using Student’s *t*-test or a Mann–Whitney test for two-independent samples.

All statistical analysis was performed in R software, version 4.4.0 [51].

All statistical tests were considered as two-sided tests, with a significant difference obtained at *p* < 0.05.

## 3. Results

The demographic and clinical characteristics of infertile women with RIF and the control group women are presented in Table 2. The mean age of the RIF group differed significantly from the mean age of the control group women (*p* = 0.0045). The patients’ mean duration of infertility and hormone levels (progesterone, luteinizing hormone, and anti-Müllerian hormone) prior to the first intervention were significantly different (Table 2).

### 3.1. Association Between the VEGF–936C/T, VEGF–634C/G, and VEGF–2578C/A Gene Polymorphisms and Recurrent Implantation Failure

The genotype distributions of VEGF–*936C/T*, VEGF–*634C/G*, and VEGF–*2578C/A* gene polymorphisms in RIF patients and the control group were presented in Table 3. Although the current study is a small study, the frequencies of the observed genotypes for each gene polymorphism in the control group were consistent with the Hardy–Weinberg equilibrium (HWE *p*-value > 0.05). A significant association was found between the odds of RIF and the variant genotype of the VEGF–*936C/T* polymorphism in the dominant model (*p* = 0.0383), patients with the (C/T+T/T) genotype having a 2.70-fold increased odds of recurrent implantation failure (adjusted-OR = 2.70, 95% CI: [1.04, 7.00]) compared to the C/C genotype. The VEGF–*2578C/A* gene polymorphism was significantly associated with an increased odds of recurrent implantation failure in the codominant model (adjusted-OR = 5.28, 95% CI: [1.42, 19.65]) and recessive model (adjusted-OR = 5.15, 95% CI: [1.55, 17.09]).

There was a significant difference in the allele distributions of VEGF–*936C/T* polymorphism between infertile women with RIF and the control group, the frequency of the *T936* allele being higher than that for the control group (28.0% vs. 14.8%) and demonstrated a 2.25-fold increased odds of implantation failure (OR = 2.25, 95% CI: [1.05, 4.81], *p* = 0.034). Similarly, we found a significant difference in the allele frequency of the VEGF–*2578C/A* gene polymorphism; the frequency of the *A2578* allele in the RIF group (53.7% vs. 33.0%) was higher than in the control group (OR = 2.36, 95% CI: [1.27, 4.39], *p* = 0.006).

### 3.2. Association of Haplotypes of VEGF Gene Polymorphisms with the Odds of RIF

The VEGF gene polymorphisms were in weak pairwise linkage disequilibrium both in the RIF group (D’_VEGF-*936/-634*_ = 0.51, *p* = 0.00031, D’_VEGF-*634/-2578*_ = 0.02, *p* = 0.9375, D’_VEGF-*936/-2578*_ = 0.24, *p* = 0.2126) and the control group (D’_VEGF-*936/-634*_ = 0.18, *p* = 0.1420, D’_VEGF-*634/-2578*_ = 0.03, *p* = 0.9197, D’_VEGF-*936/-2578*_ = 0.54, *p* = 0.1366).

The results of haplotype-based regression applied to identify three-locus haplotypes associated with the odds of RIF, in which haplotype VEGF-*936/-634/-2578 C-C-C* was chosen as a reference, revealed that the odds of RIF was 12.39 (*p* = 0.0241) in carriers of the VEGF-*936/-634/-2578* T-C-A haplotype. After adjusting for patients’ age, the odds of RIF was 8.51 (*p* = 0.0582), without statistical significance. Concerning two-locus haplotypes, the regression analysis highlighted that the VEGF-*936/-2578 T-A* haplotype had 12.23-fold (*p* = 0.0107) increased odds of RIF. The risk remained significantly increased, 9.56 (*p* = 0.0113), after adjusting for patients’ age (Table 4).

### 3.3. Comparisons of Hormones Values by Genotypes of VEGF Gene Polymorphisms

In the RIF group, we did not find any significant difference in the distribution of hormone levels between patients with the variant genotypes of studied polymorphisms and those with the wild genotype (*p* > 0.05), while in the control group, there was a significant association between VEGF–*634C/G* genotypes and testosterone (*p* = 0.0167). We noticed that patients carrying a variant (CG+GG) genotype of VEGF–*634C/G* gene polymorphism had a lower level of testosterone than patients carrying a wild (CC) genotype (median [IQR]: 0.3 [0.20, 0.4] vs. 0.5 [0.3, 1.1]). In the same group (control group), we noticed the higher values of FSH in patients carrying a variant (CG+GG) genotype of VEGF–*634C/G* gene polymorphism than patients with a wild genotype (median [IQR]: 7.5 [6.9, 9.5] vs. 6.8 [6.1, 8.1]), but the difference had only tendency toward statistical significance (Table 5).

## 4. Discussion

Recurrent implantation failure encompasses a multitude of etiologies. When contrasted with typical IVF patients, the endometrium stands out as a notably significant factor, in addition to potential paternal or oocyte quality concerns. Endometrium regeneration is governed by a myriad of molecular and cellular interactions, regulated by endocrine factors, locally produced steroids, immune factors, angiogenetic factors, and factors associated with uterine microbiota.

At the core of this intricate process are two fundamental elements: epithelial and stromal cellular maturation, and vascular remodeling. The coordinated interaction between these components drives the dynamic restructuring of the endometrial tissue, ultimately facilitating its receptivity (Cimadomo et al., 2021) [3]. The influence of angiogenesis on endometrial functionality is profound and extensively documented. Any disruptions in this process, whether excessive or deficient angiogenesis, can have significant implications for fertility (Don et al., 2023) [52].

A thin endometrium may arise from iatrogenic insults causing hypovascularization, as well as from idiopathic suboptimal angiogenesis (Kasius et al., 2014) [53]. Conversely, an excess of angiogenesis can induce endometrial dysfunction and promote estrogen-dependent conditions such as adenomyosis, leiomyoma, and polyps, each with the potential to reduce endometrial receptivity (Don et al., 2023) [52].

Commencing with the reconstruction of the vascular system through the promotion of endometrial angiogenesis, and Vascular Endothelial Growth Factor (VEGF) concurrently influences the adhesion ability of endometrial epithelial cells. Additionally, it orchestrates the secretion of numerous cytokines crucial for embryo implantation, including integrin and Leukemia Inhibitory Factor (LIF) (Guo et al., 2021) [6]. Furthermore, VEGF exhibits functional relevance in immune regulation. It acts as a modulator, mediating the immune tolerance of the maternal immune system through mechanisms such as monocyte activation or recruitment and activation of macrophages [6]. Finally, VEGF also plays a role in the late stages of folliculogenesis, contributing to the development of the perifollicular capillary network and hormone production by the corpus luteum (Devesa et al., 2019) [54].

In this study, we tried to analyzed the association between three genetic polymorphisms, *936C/T*, *634C/G*, and *2578C/A*, in the VEGF gene and the risk of RIF in Romanian infertile women. To the best of our knowledge, this is the first study performed in Romanian infertile women with RIF that analyzed these three variants in the VEGF gene. In our study, infertile women with RIF were significantly older than women in the control group (women with minor infertility), and no significant statistical differences between the two groups regarding BMI and smoking habits were found. There are statistical significant differences regarding hormone levels, testosterone, LH, and AMH between infertile women with RIF and the control group. Although significant differences in terms of hormonal values were noted between the two groups, they fell within the expected range of outcomes. Patients with minor infertility issues exhibited a notably higher prevalence of PCOS compared to those with RIF. As a result, elevated levels of AMH, testosterone, and LH were observed in the former group in comparison to the latter. Additionally, within the RIF group, a notable proportion of patients exhibited diminished ovarian reserve, leading to a slight decrease in LH levels.

The VEGF gene is polymorphic; different genetic variants located in the promoter region of the VEGF gene could altered VEGF expression and levels. There are studies which investigated polymorphisms located in the VEGF gene in different populations, one of these being performed by Chloe et al. (2018). They analyzed three polymorphisms in the VEGF gene, including -*2578C/A* (rs699947) and -*1154G/A* (rs 1570360) [55]. Regarding the association of these polymorphisms with RIF, the results are still controversial (Turienzo et al., 2020; Shim et al., 2018) [40,56].

From this point of view, Turienzo et al. (2020) analyzed different genetic model and found that the *1154G/A* polymorphism in dominant model (GG vs. GA/AA) represents an increased risk factor for RIF, the odds being 1.842 (CI 95% 1.002–3.422). A previous report performed by Goodman et al. (2008) showed higher frequency for the homozygous genotype in women with RIF as compared with the control women, the results being statistically significant (*p* = 0.02) [28]. In 2015, Vagnini et al. investigated Brazilian women and found a 2.12 (*p* = 0.01) increased risk to develop RIF in carriers of the *1154G/A* variant in the dominant model [57]. On the other hand in 2021, Zeng et al. published a meta-analysis and confirmed the association of the *1154G/A* polymorphism and RIF under the allele and dominant model; the results were statistically significant (*p* = 0.01) [34].

There are many studies which investigated the involvement of other polymorphisms located in the VEGF gene (*460T/C*, *583C/T*, *7C/T*, *405G/C*) in the ethiopathogeny of RIF, such as the studies performed by Jung et al. (2016) and Boudjenah et al. (2012) [36,58].

Regarding the *2578C/A* polymorphism, the study released in 2023 by Mrozikiewicz et al. showed that this polymorphism did not represent risk factor for RIF when they compared women with RIF and control women. But when they compared infertile women with RIF and without RIF, the authors found that this polymorphism represents a risk factor of RIF under dominant (OR = 2.34; 95% CI, 1.11–4.94, *p* = 0.023, padj. = 0.022) and long-additive (OR = 0.65; 95% CI 0.43–0.99, *p* = 0.040, padj. = 0.038) models (Mrozikiewicz et al., 2023) [59].

Moreover, Shim et al. (2018) did not confirmed the association of RIF with *1154G/A* polymorphism, but, in the same study, they showed that homozygous carriers for the -*2578C/A* polymorphism and also carriers of the -*634G* allele had increased risk to develop RIF [56]. Carriers of the *2578C/A* and *634C/G*, respectively, polymorphisms in the recessive model had 2.77 (*p* = 0.047) and 2.1 (*p* = 0.024) increased risk to develop RIF [56].

In the present study, the analysis of the VEGF-*936C/T*, VEGF-*634C/G*, and VEGF-*2578C/A* polymorphisms revealed significant differences regarding the genotype distributions in infertile women with RIF and the control women for the first two polymorphisms.

The genotyping of the VEGF-*936C/T* polymorphism showed different distributions not only of the genotypes, but also of the alleles in both groups. In our study, the frequency of *T936* alleles and TT genotypes were more higher in the RIF group than in controls. We also performed a logistic regression analysis using a different model of inheritance to evaluate whether these polymorphisms were independently risk factors for RIF. We found that the VEGF-*936C/T* polymorphism in the dominant model (CC vs. CC + CT) showed a higher prevalence in the RIF group as compared with controls (48.8% vs. 25%). The presence of this polymorphism could have been responsible for the implantation failure, the odds of developing implantation failure being 2.7 (*p* = 0.0383) after being adjusted for age and also 2.08 (*p* = 0.034) in carriers of the *T936* allele. Our results are different than that presented by Shim et al. (2018) [56].

Regarding the VEGF-*634C/G* polymorphism, in the present study, no significant difference in the prevalence of it in both groups was found. Additionally, in the RIF group, the *GG* genotype of the VEGF-*634C/G* polymorphism was slightly less frequent than in the control group. Our results did not confirm the results conducted by Shim et al. (2018), which demonstrated an association of this polymorphism with infertility [56].

Our results confirm that, for the VEGF-*2578C/A* polymorphism, the *A2578* allele and *AA* genotype were more frequent in RIF women as compared with controls (36.6% vs. 11.4%), with higher odds for RIF in the codominant (OR 5.28, *p* = 0.074) and recessive models (OR 5.15, *p* = 0.041). Also, carriers of the *A2578* allele had 2.36 increased risk to develop RIF. The logistic regression analysis showed that this polymorphism was independently associated with the odds of RIF after adjusting by age *p* = 0.0041). The results suggest the possible involvement of this polymorphism in the ethyology of RIF. In fact, Shim et al. (2018) and Mrozikiewicz et al. (2023) obtained the same results [56,59].

In this study, we used three genetic variations in the VEGF gene (*936C/T*-*634C/G*-*2578C/A*) and we tried to establish different haplotypes as markers for RIF. We found that the VEGF-*936/-634/-2578 T-C-A* three-locus haplotype was significantly associated with increased odds of RIF, but, after adjusting for patients’ age, the *T-C-A* haplotype had a marginal significance. Concerning two-locus haplotypes, the regression analysis highlighted that the VEGF-*936/-2578 T-A* two-locus haplotype was also a significant risk factor for RIF. The risk remained significantly after adjusting for patients’ age. Our results are in agreement with the results obtained in other studies regarding the odds of RIF in association with VEGF haplotypes. Shim et al. (2018) found that -*2578C/-1154G/-634G/936C*, -*2578C/-1154G/-634G/936T* and -*2578A/-1154A/-634G/936C* haplotypes could be genetic markers of RIF [56]. The risk to develop RIF were 2 (*p* = 0.006), 2.38 (*p* = 0.053) and 2.11 (*p* = 0.021) in association with these haplotypes [56]. In 2003, Lambrechts et al. (2003) reported that the VEGF-*2578/-1154/-634 A-A-G* and VEGF-*2578/-1154/-634 A-G-G* haplotypes decreased the expression and plasma concentrations of the VEGF [60].

According to Lambrechts et al. (2003) and Shahbazi et al. (2002), the presence of the *C2578* allele determine increased VEGF secretion, so reduced angiogenesis and increased risk to develop RIF could appear in carriers of the *A2578* allele [60,61]. Moreover, in position 2549 of the VEGF gene, there is a deletion/insertion of 18bp, which is in linkage with VEGF-*2578C/A* polymorphism. The presence of the deletion in this position is associated with a 1.95-fold increased transactivation. Regarding the VEGF-*634C/G* variant, this is located in myeloid zinc finger-1 (MZF1), where the MZF1 binding site is substituted for the Pax2 [60,61].

The literature reported controversial results regarding the association between decreased VEGF levels and the VEGF-*634C/G* polymorphism. So, Hansen et al. (2010) [44] gave this role to the *C634* allele. On the contrary, Wongpiyabovorn et al. (2011) and Awata et al. (2002) [43,45] showed that decreased VEGF levels are the result of the presence of *634G* allele. Maybe it is interesting to study the association of this polymorphism with others presented in the promoter region, not only with *2578C/A* variant, as we investigated in our study. Also, it will be interesting to investigate polymorphisms presented outside the VEGF gene.

When we analyzed the association between hormones levels and the polymorphisms of the VEGF gene, our results confirmed that, in the RIF group, there were no association between the three genetic variants, *936C/T*, *634C/G*, and *2578C/A*, and serum hormones levels. On the contrary, in the control group, patients carrying at least one *G634* allele had lower testosterone and higher FSH levels than carriers of the *C634* allele. There are studies which investigated the association between hormone levels and VEGF polymorphisms in RIF females. In their study, in a Korean female population with infertility, Shim et al. (2018) [56] found lower prolactin level in women carrying the *634GG* and also the *634G* allele as compared with women carrying the *634CC* or *634C* alleles. They did not find an association between other genetic polymorphisms in the VEGF gene and FSH, LH, or estradiol levels [56]. The study performed by Ben et al. (2016), which evaluated women with polycystic ovary syndrome, revealed an association between VEGF-*936C/T* polymorphism and prolactin levels, which is an angiogenic factor [62].

It has become increasingly evident that identifying significant alterations at the follicular or endometrial level specific to RIF patients requires the application of advanced molecular tools. In particular, regarding endometrial receptivity, numerous biomarkers have been proposed. These include ultrasound markers such as endometrial thickness and pattern, Doppler signs, and isolated markers obtained through biopsy. Primarily, molecular tools have been developed, with the endometrial receptivity array (ERA) being the most extensively studied. More recent variants, such as the ER Map, ER Peak, and BeReady Test, have also emerged. However, despite the potential efficacy of these tools, ongoing debates exist regarding their effectiveness, and further studies are required to more accurately define their roles and applications in assessing endometrial functionality [63].

In this context, our findings strongly indicate that infertile patients displaying a specific genotype for VEGF polymorphisms may constitute a subgroup at heightened risk for RIF. Acknowledging this connection would warrant a more aggressive treatment strategy for these individuals. Furthermore, if our results are validated by larger studies, we can advance our understanding of the pathogenetic pathways linked to VEGF, paving the way for more targeted treatment strategies.

Our study has several limitations. First, we enrolled, in this convenience sample, only 41 patients in RIF group and 41 patients in the control group with minor infertility. It is necessary to increase the patient number. Second, we investigated the role of three genetic polymorphisms in the VEGF gene, two in the promoter region and the other one in the 5′untranslated region, on the development of RIF. It is necessary, to investigate the association with other genetic variants located in the promoter region or outside of the VEGF gene. Third, we did not evaluate the VEGF level and the association of it with different VEGF variants. Due to the aforementioned limitations, further studies with a larger sample size, and also more polymorphisms, should be carried out in order to confirm the role of the VEGF gene in women with infertility. We have to study other genetic markers in association with biochemical markers and clinical characteristics in order to contribute to the diagnostics and treatment of RIF.

From a clinical perspective, one of the primary challenges practitioners face in managing infertility is time. Implementing a genetic panel that includes specific genes commonly associated with severe infertility could help identify individuals with diminished fertility potential. This strategy would facilitate a more targeted and aggressive treatment approach for patients concerned about their fertility.

Additionally, this genetic panel could identify individuals at risk for developing serious conditions, such as endometriosis or adenomyosis. A delay in diagnosis often exacerbates clinical symptoms, underscoring the importance of early detection for effective intervention.

## 5. Conclusions

We found an association between VEGF-*936C/T* and VEGF-*2578C/A* polymorphisms and the odds of RIF in this cohort of Romanian infertile women. We did not find any association between RIF and the other VEGF polymorphism, *634C/G*. Haplotype analysis suggested the role of VEGF-*936/-634/-2578 T-C-A* and VEGF-*936/-2578 T-A* haplotypes as a risk factors for RIF in females at reproductive age. The results reveal an association between the VEGF-*634C/G* polymorphism and serum FSH and LH levels.

## Figures and Tables

**Table 1:** PCR-RFLP reagents and conditions for genotyping and enzymatic digestion methods.

Sequences of Primers	Conc. of Primers (μM)	MgCl_2_ (mM)	Anneal. Temp. (C)	PCR (bp)	Restriction Enzyme/ Digest. Temp./Time	Alleles
**VEGF-*936C/T***						
FW: 5′-AAGGAAGAGGAGACTCTGCGCAGAGC-3′ RV: 5′-TAAATGTATGTATGTGGGTGGGTGTGTCTACAG-3′	0.3	2.0	68.2	208	*NlaIII*/37 °C/3 h	*C936*: 208 bp *T936*: 122, 86 bp
**VEGF-*634C/G***						
FW: 5′-ATTTATTTTTGCTTGCCATT-3′ RV: 5′-GTCTGTCTGTCTGTCCGTCA-3′	0.4	2.0	53.7	304	*BsmFI*/65 °C/1 h	*C634*: 304 bp *G634*: 193, 111 bp
**VEGF-*2578C/A***						
FW: 5′-GGATGGGGCTGACT AGGTAAGC-3′ Rv: 5′-AGCCCCCTTTTCCT CCAAC-3′	0.2	2.0	67.2	324	*BglII*/37 °C/3 h	*C2758*: 324 bp *A2758*: 202, 122 bp

FW—forward; RV—reverse; Conc.—concentration; Anneal. Temp.—annealing temperature; bp—base pair; Digest. temp.—digestion temperature.

**Table 2:** Demographic and clinical characteristics of women with and without RIF.

Characteristics	Control Women (*n*_1_ = 44)	Infertile Women with RIF (*n*_2_ = 41)	*p*-Value
Age, years ^(1)^	33.16 (4.03)	35.51 (3.33)	0.0045 *
BMI, kg/m^2 (1)^	23.09 (1.40)	23.51 (1.79)	0.2287
Smoking, *n* (%) ^(3)^	6 (13.6)	5 (12.2)	0.8432
Previous implantation failures ^(2)^	-	4 [3, 4]	^-^
Infertility diagnosis, *n* (%) ^(3)^			0.0451 *
Male	7 (15.9)	10 (24.4)	
Idiopathic	14 (31.8)	17 (41.5)	
Trompe	9 (20.5)	11 (26.8)	
PCOS	14 (31.8)	3 (7.3)	
Infertility duration, years ^(1)^	3.16 (1.95)	6.05 (1.75)	<0.0001 *
Thickness of endometrium ^(1)^	8.95 (1.65)	9.36 (2.36)	0.3613
Endometrium volume ^(2)^	4.10 [3.10, 6.13]	4.68 [3.20, 5.10]	0.8741
Severe menorrhagia, *n* (%) ^(3)^	3 (6.8)	7 (17.1)	0.1858
Severe dysmenorrhea, *n* (%) ^(3)^	6 (13.6)	13 (31.7)	0.0457 *
Adenomyosis, *n* (%) ^(3)^	14 (31.8)	16 (39.0)	0.4872
Testosterone (ng/mL) ^(2)^	0.40 [0.20, 0.68]	0.30 [0.20, 0.40]	0.0550
Progesterone (ng/mL) ^(2)^	0.30 [0.20, 0.40]	0.30 [0.20, 0.30]	0.0487 *
FSH (mUI/mL) ^(2)^	7.10 [6.20, 8.15]	7.30 [6.20, 8.10]	0.6036
LH * (mUI/mL) ^(2)^	6.70 [6.20, 7.33]	5.60 [5.10, 6.80]	0.0008 *
Estradiol (ng/mL) ^(2)^	63.00 [54.50, 72.00]	59.00 [46.00, 72.00]	0.1956
AMH (ng/mL) ^(1)^	2.07 (1.08)	1.51 (1.01)	0.0144 *

RIF = recurrent implantation failures; PCOS = Polycystic Ovary Syndrome; FSH = follicle-stimulating hormone, LH = luteinizing hormone; AMH = anti-Müllerian hormone; data presented as ^(1)^ arithmetic mean (sample standard deviation) or ^(2)^ median [25th percentile, 75th percentile] or ^(3)^ *n* = number of subjects; *p*-values obtained from Student-*t* test on independent samples, Mann–Whitney test *t* for quantitative variables, Chi-squared test or Fisher’s exact test for nominal variables; * significant result: *p*-value < 0.05.

**Table 3:** Distributions of the genotype frequencies of the studied polymorphisms between the RIF group and the control group.

Genotypes	Control Group (*n_1_* = 44)	RIF Group (*n_2_* = 41)	OR [95% CI]	*p*	p_FDR_^(a)^	Adjusted OR ^(b)^ [95% CI]	*p*	p_FDR_^(a)^	AIC
VEGF–*936C/T*				0.0711	0.1599		0.1059	0.2383	112.9
CC	33 (75.0)	21 (51.2)	Reference			Reference			
CT	9 (20.5)	17 (41.5)	2.97 [1.12, 7.88]			2.91 [1.05, 8.08]			
TT	2 (4.5)	3 (7.3)	2.36 [0.36, 15.31]			1.85 [0.28, 12.33]			
Dominant (CC vs. CT+TT)	11 (25.0)	20 (48.8)	2.86 [1.14, 7.15]	0.0222 *	0.0667	2.70 [1.04, 7.00]	0.0383 *	0.1149	111.1
Recessive (CC+CT vs. TT)	2 (4.5)	3 (7.3)	1.66 [0.26, 10.46]	0.5876	0.8620	1.30 [0.20, 8.40]	0.7849	0.9964	115.3
HWE *p*-value	0.2141	1.000							
VEGF–*634C/G*				0.8620	0.8620		0.9561	0.9964	117.3
CC	30 (68.2)	27 (65.9)	Reference			Reference			
CG	11 (25.0)	12 (29.3)	1.21 [0.46, 3.20]			1.06 [0.38, 2.93]			
GG	3 (6.8)	2 (4.9)	0.74 [0.11, 4.77]			0.78 [0.11, 5.39]			
Dominant (CC vs. CG+GG)	14 (31.8)	14 (24.2)	1.11 [0.45, 2.75]	0.8195	0.8620	1.00 [0.39, 2.59]	0.9964	0.9964	115.4
Recessive (CC+CG vs. GG)	3 (6.8)	2 (4.9)	0.70 [0.11, 4.42]	0.7029	0.8620	0.76 [0.11, 5.19]	0.7809	0.9964	115.3
HWE *p*-value	0.1744	0.6265							
VEGF–*2578C/A*				0.0192 *	0.0667		0.0163 *	0.0734	109.2
CC	20 (45.5)	12 (29.3)	Reference			Reference			
CA	19 (43.2)	14 (34.1)	1.23 [0.45, 3.32]			1.05 [0.37, 3.01]			
AA	5 (11.4)	15 (36.6)	5.00 [1.45, 17.27]			5.28 [1.42, 19.65]			
Dominant (CC vs. CA+AA)	24 (54.6)	29 (70.7)	2.01 [0.82, 4.94]	0.1223	0.2201	1.86 [0.73, 4.76]	0.1923	0.3461	113.7
Recessive (CC+CA vs. AA)	5 (11.4)	15 (36.6)	4.50 1.46, 13.89]	0.0054 *	0.0486	5.15 [1.55, 17.09]	0.0041 *	0.0369 *	107.2
HWE *p*-value	1.000	0.0582							

RIF = recurrent implantation failures; OR = unadjusted odds ratio; 95% CI = 95% confidence interval; ^(a)^ false-discovery-rate-corrected *p*-value for multiple hypotheses testing; ^(b)^ adjusted by age; AIC = Akaike information criterion; HWE = Hardy–Weinberg equilibrium; * significant results: *p* < 0.05.

**Table 4:** Three-locus and two-locus haplotype results for associations of VEGF gene polymorphisms with RIF risk in all samples.

Estimated Haplotypes	HF in All Sample	HF in Control Group	HF in RIF Group	OR ^(a)^ [95% CI]	*p*-Value	OR ^(b)^ [95% CI]	*p*
Three-locus haplotypes (VEGF-*936C/T*-*634C/G*-*2578C/A*)							
C-C-C	0.3841	0.4566	0.3462	Reference haplotype		Reference haplotype	
C-C-A	0.2970	0.2561	0.2988	1.07 [0.49, 2.32]	0.8683	1.05 [0.47, 2.33]	0.9031
C-G-A	0.0255	0.0251	0.0487	NA	<0.0001	NA	0.5036
C-G-C	0.0817	0.1145	0.0258	NA	NA	NA	<0.0001
T-C-A	0.0523	0.0127	0.1351	12.39 [1.39, 110.44]	0.0241 *	8.51 [0.93, 78.01]	0.0582
T-C-C	0.0725	0.0815	0.0248	NA	<0.0001	NA	<0.0001
T-G-A	0.0547	0.0357	0.0540	NA	0.7921	8.48 [0.12, 621.75]	0.3293
T-G-C	0.0323	0.0179	0.0666	NA	<0.0001	NA	<0.0001
Two-locus haplotypes							
VEGF *936C/T*-*634C/G* ^(1)^							
C-C	0.6791	0.7096	0.6500	Reference haplotype		Reference haplotype	
C-G	0.1091	0.1427	0.0695	0.39 [0.11, 1.39]	0.1479	0.32 [0.09, 1.13]	0.0772
T-C	0.1267	0.0973	0.1549	1.43 [0.50, 4.05]	0.5003	1.11 [0.38, 3.28]	0.8519
T-G	0.0850	0.0505	0.1256	3.66 [0.90, 14.89]	0.0700	4.22 [0.98, 18.12]	0.0531
VEGF *634C/G*-*2578C/A* ^(2)^							
C-C	0.4582	0.5390	0.3713	Reference haplotype		Reference haplotype	
C-A	0.3477	0.2679	0.4336	1.87 [0.96, 3.67]	0.0671	1.81 [0.90, 3.63]	0.0965
G-A	0.0817	0.0617	0.1030	2.96 [0.66, 13.37]	0.1577	3.10 [0.72, 13.31]	0.1276
G-C	0.1124	0.1315	0.0921	0.74 [0.20, 2.76]	0.6556	0.64 [0.18, 2.30]	0.4935
VEGF *936C/T*-*2578C/A* ^(3)^							
C-C	0.4611	0.5450	0.3642	Reference haplotype		Reference haplotype	
C-A	0.3271	0.3073	0.3553	1.51 [0.75, 3.03]	0.2506	1.52 [0.73, 3.14]	0.2592
T-A	0.1023	0.0222	0.1813	12.23 [1.79, 83.62]	0.0107 *	9.56 [1.67, 54.84]	0.0113 *
T-C	0.1095	0.1255	0.0992	1.14 [0.39, 3.30]	0.8129	1.10 [0.38, 3.15]	0.8603

Note: Alleles in haplotype are described in order of studied gene polymorphisms (VEGF*936C>T*,′′VEGF*634C>G* and′′VEGF*2578C>A*); global haplotype association *p*-value for additive three-locus haplotype model: 0.0176; ^(1)^ global haplotype association *p*-value for additive two-locus haplotype model: 0.0876; ^(2)^ global haplotype association *p*-value for additive two-locus haplotype model: 0.09156; ^(3)^ global haplotype association *p*-value for additive two-locus haplotype model; 0.00798; HF = haplotype frequencies; NA = not available due to absence or low frequency in one of the two groups; ^(a)^ odds ratio of each haplotype estimated from haplotype-based GLM regression without covariates; ^(b)^ odds ratios of each haplotype adjusted for age (years); 95% CI: 95% confidence interval; * significant result: *p* < 0.05.

**Table 5:** Distributions of hormone values by the genotypes of studied gene polymorphisms.

Characteristics	RIF Group			Control Group		
**VEGF*–936C/T* genotypes**	**CC (*n*_1_ = 21)**	**CT+TT (*n*_2_ = 20)**	***p***	**CC (*n*_1_ = 33)**	**CT+TT (*n*_2_ = 11)**	***p***
Testosterone (ng/mL) ^(a)^	0.3 [0.2, 0.4]	0.3 [0.2, 0.3]	0.3986	0.3 [0.2, 0.6]	0.4 [0.3, 1.1]	0.5467
Progesterone (ng/mL) ^(a)^	0.3 [0.2, 0.3]	0.25 [0.2, 0.325]	0.8278	0.3 [0.3, 0.4]	0.3 [0.2, 0.4]	0.5077
FSH (mUI/mL) ^(a)^	7.9 [6.9, 8.8]	6.6 [6.1, 7.9]	0.0948	7.1 [6.2, 8.1]	7.1 [6.3, 8.9]	0.6643
LH (mUI/mL) ^(b)^	5.4 [4.8, 6.4]	6.3 [5.2, 6.8]	0.3145	6.7 [6.3, 7.1]	7.1 [6.1, 8.6]	0.8068
Estradiol (ng/mL) ^(a)^	61.0 [49.0, 72.0]	55.5 [42.0, 73.8]	0.6665	63.0 [53.0, 72.0]	64.0 [55.0, 75.0]	0.7446
AMH (ng/mL) ^(b)^	1.4 (1.2)	1.6 (0.8)	0.5674	1.9 (1.0)	2.3 (1.3)	0.371
**VEGF*–634C/G* genotypes**	**CC (*n*_1_ = 27)**	**CG+GG (*n*_2_ = 14)**		**CC (*n*_1_ = 30)**	**CG+GG (*n*_2_ = 14)**	
Testosterone (ng.mL) ^(a)^	0.2 [0.2, 0.4]	0.3 [0.3, 0.4]	0.0707	0.5 [0.3, 1.1]	0.3 [0.2, 0.4]	0.0167 *
Progesterone (ng/mL) ^(a)^	0.3 [0.2, 0.3]	0.3 [0.3, 0.4]	0.3819	0.3 [0.3, 0.4]	0.3 [0.2, 0.38]	0.4017
FSH (mUI/mL) ^(a)^	7.4 [6.8, 8,9]	6.4 [6.1, 7.9]	0.1605	6.8 [6.1, 8.1]	7.5 [6.9, 9.5]	0.0553
LH (mUI/mL) ^(b)^	5.7 [4.9, 6.9]	5.5 [5.2, 6.4]	0.8256	6.6 [6.1, 7.1]	7.0 [6.4, 8.1]	0.1362
Estradiol (ng/mL) ^(a)^	5.7 [4.9, 6.9]	5.5 [5.2, 6.4]	0.8688	61.5 [53.5, 72.8]	64.0 [61.5, 71.3]	0.8697
AMH (ng/mL) ^(b)^	1.4 (1.1)	1.7 (0.8)	0.4374	2.3 (1.0)	1.5 (0.98)	0.0140 *
**VEGF*–2578C/A* genotypes**	**CC (*n*_1_ = 12)**	**CA+AA (*n*_2_ = 29)**		**CC (*n*_1_ = 20)**	**CA+AA (*n*_2_ = 24)**	
Testosterone (ng.mL) ^(a)^	0.3 [0.2, 0.6]	0.3 [0.2, 0.4]	0.5077	0.6 [0.2, 1.0]	0.3 [0.2, 0.4]	0.2837
Progesterone (ng/mL) ^(a)^	0.3 [0.2, 0.3]	0.3 [0.2, 0.3]	0.8227	0.3 [0.3, 0.4]	0.3 [0.2, 0.4]	0.5160
FSH (mUI/mL) ^(a)^	6.9 [5.9, 7.5]	7.9 [6.4, 8.8]	0.1215	6.9 [6.0, 8.4]	7.1 [6.4, 7.9]	0.9812
LH (mUI/mL) ^(b)^	6.0 [5.4, 6.8]	5.4 [4.9, 6.8]	0.3436	6.6 [6.3, 7.5]	6.9 [6.2, 7.3]	0.9906
Estradiol (ng/mL) ^(a)^	55.0 [45.0, 69.0]	61.0 [48.0, 79.0]	0.4910	63.0 [55.0, 78.0]	63.5 [52.0, 72.0]	0.6368
AMH (ng/mL) ^(b)^	1.6 (1.3)	1.5 (0.9)	0.7190	2.2 (1.2)	1.9 (0.9)	0.4631

RIF = recurrent implantation failures; FSH = follicle-stimulating hormone, LH = luteinizing hormone; AMH = anti-Müllerian hormone; data presented as ^(a)^ median [25th percentile, 75th percentile] or ^(b)^ arithmetic mean (sample standard deviation); *n* = number of subjects; *p*-values obtained from Mann–Whitney test or Student-t test on independent samples; * significant result: *p*-value < 0.05.

## Data Availability

The raw data involved in this study can be obtained upon reasonable request addressed to Lucia M. Procopciuc (lprocopciuc@umfcluj.ro).
